# Long-gap esophageal atresia: a single center experience

**DOI:** 10.3389/fped.2025.1566738

**Published:** 2025-05-06

**Authors:** Xiaoshu Liu, Xue Sun, Hongxia Ren

**Affiliations:** ^1^ Department of Neonatal Surgery, Shanxi Provincial Children's Hospital, Taiyuan, China; ^2^ Department of Pediatrics, Shanxi Medical University, Taiyuan, China

**Keywords:** long-gap esophageal atresia (LGEA), esophageal anastomosis, prognosis, treatment, esophageal elongation

## Abstract

**Objective:**

To summarize the treatment experience and individualized treatment strategies for children with long-gap esophageal atresia (LGEA) at a single center.

**Methods:**

The clinical data of children with LGEA admitted to Shanxi Provincial Children's Hospital from January 2018 to December 2024 were collected and analyzed. The data included classification, gap length, timing of surgery, methods of esophageal elongation, methods of esophageal anastomosis, postoperative complications, prognosis, etc.

**Results:**

A total of 7 children with LGEA were studied, with 3 males and 4 females. Among them, 6 cases were Type I esophageal atresia (EA), 1 case was Type II EA. The average distance between the blind ends of the esophagus was approximately (5.36 ± 0.75) cm. All 7 cases were followed up completely, with 5 cases achieving full recovery, 1 cases having poor prognosis, and 1 cases resulting in death. The overall mortality rate was 14.28% (1/7). Type I EA had 6 cases, with the esophageal blind ends approximately (5.25 ± 0.76) cm apart during the neonatal period. All underwent staged surgery: stage I involved gastrostomy during the neonatal period, and stage II involved esophageal anastomosis, gastric replacement esophagectomy, or colonic replacement esophagectomy. The average age at stage II surgery was (210.83 ± 115.75) days. Type II EA had 1 case, with the esophageal blind ends approximately 6 cm apart during the neonatal period. Staged surgery was performed: Stage I, gastrostomy during the neonatal period; Stage II, esophageal-tracheal fistula ligation and intra-thoracic esophageal traction at both ends; Stage III, esophageal anastomosis.

**Conclusion:**

The treatment of LGEA is still challenging, good treatment results can be obtained by formulating a personalized treatment plan, selecting an appropriate surgical method, delaying anastomosis, preserving the original esophagus as much as possible, strengthening perioperative management, and establishing long-term follow-up.

## 1 Introduction

Esophageal atresia (EA) is a lethal congenital gastrointestinal malformation in newborns, with an incidence of about 1 in 3,500. Long-gap esophageal atresia (LGEA) accounts for 8% of EA cases. The International Network for Esophageal Atresia defines LGEA as EA with no air in the abdomen. The European Reference Network on Rare Genetic Congenital Malformations considers “EA with no air in the abdomen” or “EA with a gap between the esophageal ends spanning three or more vertebrae” as diagnostic criteria for LGEA. It is generally believed that a distance of ≥3 cm between the proximal and distal esophagus can be defined as LGEA. However, there are no unified guidelines for the assessment, treatment, and management of LGEA (1–6), and many pediatric surgeons continue to explore the diagnosis and treatment of LGEA. This article summarizes the data of 7 LGEA patients admitted to our hospital over a period of 7 years, analyzes the different surgical plans and outcomes based on the varying conditions of the patients, and summarizes the treatment experience and corresponding individualized treatment strategies.

## 2 Methods

A retrospective analysis was conducted on 7 LGEA patients admitted to Shanxi Provincial Children's Hospital from January 2018 to December 2024. This study complied with the regulations of the Ethics Committee of Shanxi Medical University Affiliated Children's Hospital and was approved by the Ethics Committee (IRB-KYYN-2023-010) and the Helsinki Declaration (as revised in 2013).The clinical data of the children are shown in Table 1.

**Table 1:** Clinical data of children with LGEA.

Pt.	Type	Gap length (cm)	Age at esophageal anastomosis (d)	Methods of esophageal elongation	Surgical access	Complications	Prognosis
1	Ⅰ	5	123	(1), (2)	Thoracoscopic	Fulminant enteritis, multi-organ failure	Death
2	Ⅰ	4	61	(1)	Thoracoscopic	None	Recovery
3	Ⅰ	6	308	(1), (2), (3)	open	Hypoxic-ischemic encephalopathy, GER (pyloroplasty)	Poor (epilepsy)
4	Ⅰ	5	191	(1), (2)	Thoracoscopic	GER (Nissen surgery)	Recovery
5	Ⅰ	5.5	350	(1), (2)	Thoracoscopic	GER	Recovery
6	Ⅰ	6	273	(1), (2), (4)	open	Esophageal stenosis	Recovery
7	Ⅱ	6	362	(1), (2), (5)	Thoracoscopic	None	Recovery

Pt., patient; BW, birth weight; GA, gestational age; (1), natural elongation; (2), internal bougienage stretching technique; (3), gastric esophageal replacement; (4), colonic esophageal replacement; (5), intrathoracic traction elongation; GER, gastroesophageal reflux.

### 2.1 Inclusion criteria

(1) Patients with a preoperative examination confirming a gap length of ≥3 vertebrae or ≥3 cm were diagnosed with LGEA; (2) The proximal and distal segments of the esophagus are too far apart to enable primary anastomosis via a single operation in the newborn period; (3) Type I: isolated esophageal atresia without tracheoesophageal fistula. Type II: esophageal atresia with proximal tracheoesophageal fistula; (4) Patients without severe malformations of the heart, lungs, or kidneys before surgery and without surgical contraindications; (5) The legal guardian signed the surgical consent form, agreeing to accept the surgical method and to follow up as scheduled.

### 2.2 Observational indicators

Classification, gap length, timing of surgery, methods of esophageal elongation, methods of esophageal anastomosis, postoperative complications, prognosis, etc.

## 3 Results

**Figure 1:**
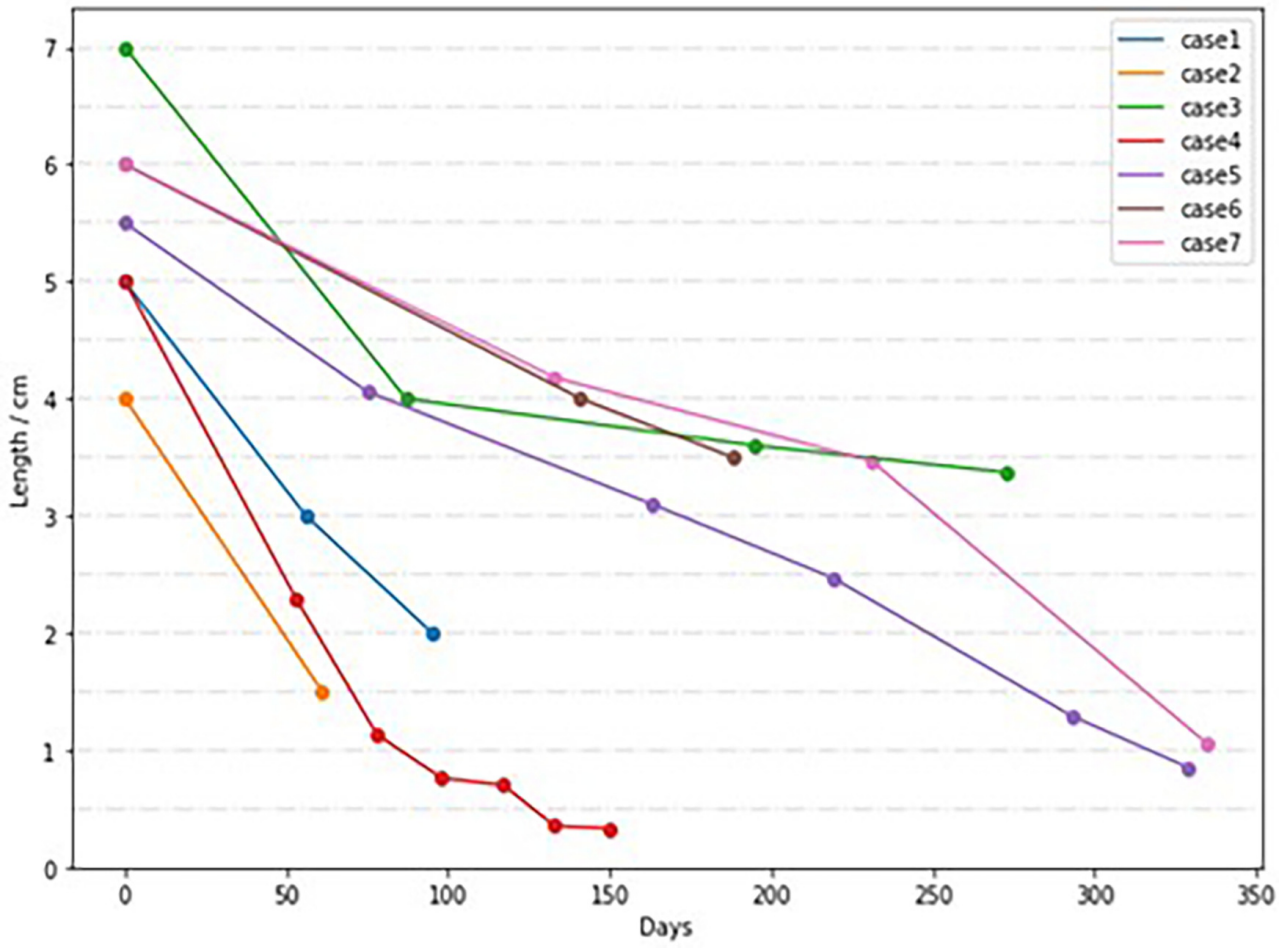
Distance between esophageal blind ends in children with delayed anastomosis at different ages.

This study collected data from 7 children with LGEA, including 4 males and 3 females. Among them, there were 6 cases of type I EA, 1 case of type II EA. The distance between the blind ends of the esophagus was approximately (5.36 ± 0.75) cm, with an average weight of (2,184.29 ± 494.30) g. The follow-up time after esophageal anastomosis ranged from 4 to 72 months. All 7 cases were followed up completely, with 5 cases achieving full recovery, 1 cases resulting in death (one infant died of fulminant enteritis and multi-organ failure half a year after the esophageal anastomosis), and 1 cases having poor prognosis (one case has poor quality of life after surgery due to epilepsy). The overall mortality rate was 14.28% (1/7, with 1 case of type I). All 7 patients underwent delayed anastomosis. The distance between the blind ends of the esophagus in children undergoing delayed anastomosis was measured by esophagography at different ages before surgery, as shown in Figure 1.

### 3.1 Typing and prognosis

#### 3.1.1 Type I EA

There were 6 cases of type I EA, with the blind ends of the esophagus approximately (5.25 ± 0.76) cm apart, all undergoing staged surgery, with the second-stage surgery age being (210.83 ± 115.75) days. The first-stage surgery involved gastrostomy, and the second-stage surgery methods included esophageal anastomosis (4 cases), gastric replacement esophagectomy (1 case), and colonic replacement esophagectomy (1 case), with no anastomotic leaks occurring postoperatively, and a mortality rate of 16.67% (1/6). Among the 6 cases, 3 recovered smoothly, 1 case developed esophageal stenosis 2 months after colonic replacement esophagectomy and underwent reanastomosis at another hospital; 1 case had poor quality of life due to epilepsy after gastric replacement esophagectomy and pyloroplasty (unrelated to the primary disease); 1 case died of fulminant enteritis and multi-organ failure half a year after the esophageal anastomosis.

#### 3.1.2 Type II EA

**Figure 2:**
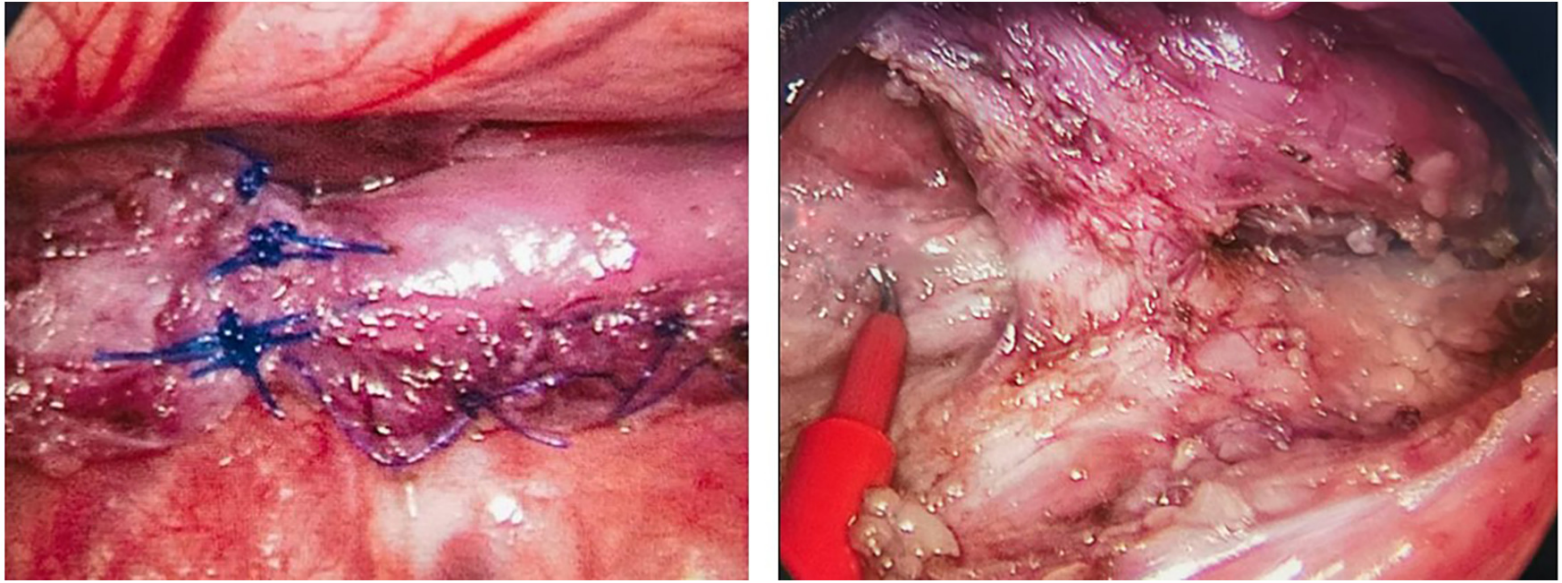
Intraoperative findings in a child with type II esophageal atresia.

There was 1 case of type II EA, with the blind ends of the esophagus approximately 6 cm apart, initially misdiagnosed as type I after admission, and underwent gastrostomy in the neonatal period; at 232 days, the second-stage surgery was performed, and it was discovered to be type II EA during the surgery, leading to esophagotracheal fistula ligation and intrathoracic traction of both ends of the esophagus. Four months later (at 362 days of age), esophageal anastomosis was performed. Due to anastomotic stenosis, esophageal dilation was performed, and currently, the gastrostomy tube is completely clamped, awaiting closure. The intraoperative findings are shown in Figure 2.

### 3.2 Long-term postoperative complications

In addition to early complications such as esophageal stenosis and anastomotic leaks, children also have long-term complications such as dysphagia and gastroesophageal reflux (GER).

#### 3.2.1 Dysphagia

All children were followed up from 4 to 72 months, with 2 children experiencing functional dysphagia and being unable to feed orally for half a year, requiring enteral feeding through a gastric tube or nasojejunal feeding tube, and were able to slowly feed orally at 7 and 8 months, respectively; 5 children were treated with omeprazole and other acid-suppressing drugs for more than 1 year after surgery.

#### 3.2.2 GER

During the follow-up period, 3 children had varying degrees of GER. Two children underwent surgery due to clinical symptoms of GER (1 underwent Nissen surgery and 1 underwent pyloroplasty), and one children was treated with positional therapy to alleviate symptoms.

## 4 Discussion

At present, most of the esophageal gap ≥3 vertebral bodies or ≥3 cm is the LGEA standard (1–5). Most scholars believe that it is more clinically significant to define EA as LGEA when primary anastomosis remains unachievable even after adequate mobilization of the esophageal ends during the neonatal period (4). The treatment of LGEA is still challenging. Not only the operation is difficult, the risk is high, the course of treatment is long, and the incidence of postoperative complications are high, but the long-term growth and development of some children will be affected, and the compliance of parents to receive long-term treatment is tested (7–9). Therefore, the decision-making of the best treatment plan for LGEA, the choice of surgical methods, the reduction of postoperative complications, the improvement of the cure effect and the improvement of the long-term quality of life have always been our concern.

### 4.1 The timing of the operation

At present, it is generally believed that the optimal natural growing time of the esophagus is during the first 8–12 weeks after birth, which is caused by swallowing reflex and reflux of gastric contents to the stimulation of the lower esophagus (10). Most scholars believe that LGEA is expected to coincide directly at the age of 3–5 months. However, it is also reported that if the initial distance reaches 7.0 cm or 8 vertebrae, the surgical anastomosis can be delayed to 12 months (11). Analyzing the data of this center, there is no unified time for the surgical anastomosis of this group, and the shortest age of the operation is 61 days and the longest is 362 days. Cases 1, 2, and 4 underwent esophageal anastomosis after natural esophageal elongation or esophageal tension elongation, at 123, 61, and 191 days, respectively; Cases 3 and 6 had poor results with esophageal self-elongation and underwent colonic replacement esophagectomy at 273 days and gastric replacement esophagectomy at 308 days, respectively; Cases 5 and 7 exhibited slow growth in the later stages and underwent esophageal anastomosis at 350 days and 362 days, respectively. It can be seen that if the distance of the blind end of the initial esophageal is ≤5 cm, the two blind ends are quickly close after 2–5 months of natural growth and internal tension traction, and the probability of completing esoesophageal anastomosis is high; if the distance of the blind end of the initial esophageal is ≥5 cm, the esophagus itself is slow in the later stage of extension, and the esophageal growth is regularly monitored, and surgery can be performed when the distance between the two ends is close. If the effect of extension is not good, replacement surgery can be performed as appropriate after the age of 6 months. The diagnosis of LGEA is synonymous with a prolonged hospitalization when compared to those without EA (12, 13). Prior to delayed anastomosis, prolonged hospitalization is typically required in this study. Gastrostomy provides access for enteral nutritional support, with adequate enteral feeding not only improving the infant's general nutritional status but also promoting growth of the esophageal pouches. Continuous low-pressure suction of the esophageal pouch helps reduce risks of aspiration and aspiration pneumonia, optimizes pulmonary status, and creates optimal conditions for subsequent surgical interventions. In a word, monitoring esophageal growth, regular comprehensive evaluation, and elective radical treatment are the treatment principles of LGEA.

### 4.2 Surgical method

LGEA surgical methods include various types of esophageal replacement surgery and esophageal extension surgery (2, 4, 6). The surgical treatment method of early LGEA is more esophageal replacement surgery. In recent years, the preservation of the primary esophagus has been advocated by more and more doctors. Postponed anastomosis and traction techniques help LGEA patients use the primary esophagus to avoid serious long-term complications related to esophageal replacement (1, 14). Some scholars have also pointed out that botulinum toxin may play a role in accelerating the traction-induced esophageal growth process for LGEA repair (15). The Chinese consensus states that if the medical unit has a high level of NICU monitoring and nursing, the distance between the esophageal blind end is located between 2 and 6 vertebraes, and the parents' economic tolerance is good, the postponement of anastomosis surgery can be tried on a trial (16).

In this study, cases 1, 2, 4, 5 and 7 performed deferred esophageal anastomosis; cases 3 and 6 had poor effect on the esophagus itself, and colonic esophageal replacement and gastric esophageal replacement were performed respectively. It can be seen that if the distance of the blind end of the initial esophagus is ≤5 cm, the two blind ends are quickly approaching after 2–5 months of natural growth and internal tension traction, and the probability of completing esophageal anastomosis is high; if the distance of the initial esophageal blind end is ≥5 cm, the early effect of self-extension in the esophagus is greater than the later stage, and certain treatment can be achieved through intrathoracic traction, those with poor effect need to undergo esophageal replacement surgery; The optimal timing for intrathoracic traction should be individualized, taking into comprehensive consideration of the child's anatomical characteristics, comorbidities, developmental status, and surgical conditions. Generally, traction is maintained for 6–12 weeks, but in cases of extremely long gaps (>5 cm), the traction period may need to be extended to 12–16 weeks or longer, requiring a tailored approach based on clinical progression and tissue adaptability. Our center has limited experience with traction (case 7 with a total traction duration of 130 days), and further research is needed to refine the optimal timing and duration of traction; gastrostomy can make the child enteral nutrition as soon as possible, and escort nutritional support and as soon as possible healing of the anastomosis; in addition, for children with type II esophageal atresia (the proximal esophagus is connected to the trachea), the risk of complications of esophageal anastomosis, esophageal trachea truction and suture fistula in stage II at the same time is relatively high. For the sake of insurance, it is a safe surgical method to ligation of the fistula first, traction of the two-end esophagus first, and then esophageal anastomosis at a time. Therefore, there are various treatment options for LGEA that can be individualized according to the specific situation of the patient, and delayed anastomosis and preservation of the original esophagus should be the first choice.

### 4.3 Postoperative complications

It has been reported that LGEA will inevitably have some long-term complications, including GER (95%) and difficulty swallowing (9, 17, 18).

#### 4.3.1 Dysphagia

The survival rate of LGEA patients is about 90%. It is reported that most patients can eat normally, and the incidence of difficulty swallowing and food aversion is 21%–84%. Some patients can cover up their symptoms by adjusting their own diet, which is often found at presentation mostly due to malnutrition and poor growth (3, 9, 17–19). Difficulty in swallowing is mainly mechanical and functional. Mechanical dysphagia is most commonly caused by anastomotic stricture and can be managed with straightforward interventions. Multiple studies have identified delayed anastomosis, long-gap defects, and GER as risk factors for postoperative anastomotic strictures (20–22), with most cases improving through regular dilatation. Longer stricture length and smaller luminal diameter correlate with an increased number of dilatations required for resolution (23). Early identification of strictures and prompt endoscopic intervention significantly improve outcomes. Therefore, routine contrast studies should be performed every 3–4 weeks post-anastomosis to detect strictures, followed by timely endoscopic esophageal dilatation. Gradual dilatation should continue until mucosal tearing occurs, achieving a luminal diameter ≥5 mm (23). In this study, all 7 cases underwent regular dilatation, with only one patient requiring reoperation for refractory stricture. All patients currently demonstrate age-appropriate growth and development. Functional dysphagia has complex causes and is not easy to deal with. In this study, 2 cases of functional dysphagia were not combined with esophageal stenosis, indicating that esophageal stenosis is only one of the causes of dysphagia, and there may also be some physiological structural changes that cause postoperative dysphagia. Most of them can be reduced or recovered after swallowing training (16, 19). Clinical swallowing assessment (CSE) is an effective technique to improve feeding results and can quickly achieve full oral feeding (24).

#### 4.3.2 GER

GER is a common problem in EA patients, with an incidence rate of 30%–70%. The risk of gastroesophageal reflux in children with LGEA is higher (5, 18, 19, 25–27). GER can often be managed with conservative therapy. When conservative measures fail, the timing of anti-reflux surgery requires individualized decision-making. Current GER management primarily relies on pharmacological treatment, while surgical and endoscopic interventions are reserved for specific scenarios (28). However, controversies persist regarding optimal surgical timing, the choice between surgical and endoscopic approaches, personalized treatment strategies, and long-term outcome evaluation. We propose the following surgical indications: Failure of adequate-dose pharmacotherapy (e.g., omeprazole 1–2 mg/kg/day) for ≥3 months; Persistent symptoms impairing growth and development; Postoperative GER persisting beyond 6 months; Severe respiratory complications secondary to reflux. In our cohort of 7 cases, 3 patients exhibited GER symptoms. All received conservative management, including positioning therapy, continuous pump feeding, and omeprazole for acid suppression. 2 cases showed poor response to conservative measures: one underwent Nissen fundoplication combined with gastrostomy to address reflux and feeding challenges; one underwent pyloroplasty to enhance gastric emptying and reduce reflux risk from elevated intragastric pressure. Our approach aligns with existing research while introducing more specific clinical indicators, aiming to provide novel insights for optimizing GER management in similar cases. The data in this group lacks objective inspection methods such as esophageal pH monitoring, and the case follow-up time is relatively short. Barrett's metasification and other conditions have not been found, so long-term follow-up attention is needed (3, 17).

## 5 Conclusions

The treatment of LGEA is still challenging, and good treatment results can be obtained by formulating a personalized treatment plan, selecting an appropriate surgical method, delaying anastomosis, preserving the original esophagus as much as possible, strengthening perioperative management, and establishing long-term follow-up.

## Data Availability

The original contributions presented in the study are included in the article/Supplementary Material, further inquiries can be directed to the corresponding author.
